# Direct metagenomics investigation of non-surgical hard-to-heal wounds: a review

**DOI:** 10.1186/s12941-024-00698-z

**Published:** 2024-05-03

**Authors:** Madjid Morsli, Florian Salipante, Chloé Magnan, Catherine Dunyach-Remy, Albert Sotto, Jean-Philippe Lavigne

**Affiliations:** 1 Department of Microbiology and Hospital Hygiene, VBIC, INSERM U1047, Univ Montpellier, Platform MICRO&BIO, CHU Nîmes, Nîmes, France; 2 Department of Biostatistics, Clinical Epidemiology, Public Health, and Innovation in Methodology (BESPIM), CHU Nîmes, Nîmes, France; 3 Department of Infectious Diseases, VBIC, INSERM U1047, Univ Montpellier, CHU Nîmes, Nîmes, France

**Keywords:** Non-surgical hard-to-heal wounds, 16S rDNA metagenomics, Shotgun metagenomics, microbial diversity, Wound-colonising microorganisms, Wound healing, Pathogen genome detection

## Abstract

**Background:**

Non-surgical chronic wounds, including diabetes-related foot diseases (DRFD), pressure injuries (PIs) and venous leg ulcers (VLU), are common hard-to-heal wounds. Wound evolution partly depends on microbial colonisation or infection, which is often confused by clinicians, thereby hampering proper management. Current routine microbiology investigation of these wounds is based on in vitro culture, focusing only on a limited panel of the most frequently isolated bacteria, leaving a large part of the wound microbiome undocumented.

**Methods:**

A literature search was conducted on original studies published through October 2022 reporting metagenomic next generation sequencing (mNGS) of chronic wound samples. Studies were eligible for inclusion if they applied 16 S rRNA metagenomics or shotgun metagenomics for microbiome analysis or diagnosis. Case reports, prospective, or retrospective studies were included. However, review articles, animal studies, in vitro model optimisation, benchmarking, treatment optimisation studies, and non-clinical studies were excluded. Articles were identified in PubMed, Google Scholar, Web of Science, Microsoft Academic, Crossref and Semantic Scholar databases.

**Results:**

Of the 3,202 articles found in the initial search, 2,336 articles were removed after deduplication and 834 articles following title and abstract screening. A further 14 were removed after full text reading, with 18 articles finally included. Data were provided for 3,628 patients, including 1,535 DRFDs, 956 VLUs, and 791 PIs, with 164 microbial genera and 116 species identified using mNGS approaches. A high microbial diversity was observed depending on the geographical location and wound evolution. Clinically infected wounds were the most diverse, possibly due to a widespread colonisation by pathogenic bacteria from body and environmental microbiota. mNGS data identified the presence of virus (EBV) and fungi (*Candida* and *Aspergillus* species), as well as *Staphylococcus* and *Pseudomonas* bacteriophages.

**Conclusion:**

This study highlighted the benefit of mNGS for time-effective pathogen genome detection. Despite the majority of the included studies investigating only 16 S rDNA, ignoring a part of viral, fungal and parasite colonisation, mNGS detected a large number of bacteria through the included studies. Such technology could be implemented in routine microbiology for hard-to-heal wound microbiota investigation and post-treatment wound colonisation surveillance.

**Supplementary Information:**

The online version contains supplementary material available at 10.1186/s12941-024-00698-z.

## Introduction

Non-surgical chronic wounds constitute a significant number of non-healing or delayed-healing wounds [1–3]. The Wound Healing Society defines four types of chronic wounds: diabetes-related foot diseases (DRFD), vascular ulcers (venous and arterial ulcers), and pressure injuries (PI) [4, 5]. DRFDs are particularly prevalent in uncontrolled diabetes mellitus, increasing the risk of diabetic foot infection (DFI), which can progress to diabetic foot osteomyelitis (DFOM) and have a significant impact on the morbidity and mortality of this population [6]. Indeed, the severity of DFI evolution leads to foot amputations and mortality in 17% and 15% of cases, respectively [7, 8]. PIs are unrelieved injuries caused by sustained soft tissue compression bordering the bones, classified into six stages according to the National Pressure Injury Advisory Panel depending on the severity of the PI and its evolution [9, 10]. This may progress to life-threatening complications in 21–58% of PIs, including 27% of recurrent wounds [11]. Venous leg ulcers (VLU) represent from 60 to 80% of all lower-limb ulceration [12]. The risk of VLU increases among older people with concomitant chronic venous insufficiency [12]. In all these chronic wounds, patient care is expensive, challenging clinical management and wound healing [1–3, 13, 14].

Chronic wounds healing is usually impacted by colonising microorganisms [15–17]. Skin, digestive and/or environmental microbiomes are the main origins of the microorganisms colonising the wounds, while hospital environment including medical equipment and healthcare professionals increases the risk of cross-contamination and colonisation by multidrug resistant microorganisms [3, 18–21]. However, the distinction between colonisation by normal opportunistic microorganisms and infection due to pathogenic bacteria remains a challenge in clinical microbiology, and incorrect diagnosis contributes to delayed wound management and patient cure [17, 20]. Current routine microbiological investigation of chronic wounds using swabs and/or tissue biopsies are mainly based on in vitro culture inoculation, limiting the discoverable bacteria [14]. *Staphylococcus aureus* is the most prevalent Gram-positive bacteria identified in routine bacteriology, including a high rate of methicillin resistance [22]. Other *Staphylococcus* species, such as *Staphylococcus epidermidis* potentially transmitted from skin microbiota, and other bacteria belonging to the Firmicutes phylum (e.g., *Streptococcus agalactiae*, *Streptococcus pyogenes*, *Streptococcus mitis*, and *Enterococcus faecalis*) are also frequently identified in these clinical situations [23]. Enterobacteriaceae family including *Escherichia, Klebsiella*, *Enterobacter, Citrobacter*, *Proteus* and *Serratia* species and non-fermenting Gram-negative bacilli including *Pseudomonas* and *Stenotrophomonas*, are the predominant Gram-negative bacteria in chronic wounds, particularly frequent in PIs, chronic and recurrent DFU and in warm countries [14, 22].

Routine molecular detection tools of chronic wounds infections are based on simplex or multiplex real-time PCR targeting a limited number of bacteria commonly isolated from chronic wound samples, as well as the partial sequencing of the 16 S rDNA [23, 24]. The lack of universal identification of microorganisms involved in these infections complicates diagnosis and patient management. Moreover, the polymicrobial biofilm formation present in most chronic or hard-to-heal wounds challenges the antimicrobial therapy. The secretion of immune evasion factors increases and extends the inflammation response, delaying the wound healing [25, 26]. Metagenomic next generation sequencing (mNGS) of chronic wound swabs and biopsies targeting 16 S rDNA gene have emerged within the last 15 years [27]. Shotgun mNGS has been developed to detect and characterise mono- and polymicrobial infections in record time. Based on the limited molecular findings in accordance with clinical opinions qualifying microorganisms infecting or colonising the wounds, current international consensus suggests that molecular techniques should not be used for the first-line identification of pathogens from tissue or bone samples in a patient with a DFI [8, 28]. To our knowledge, no papers have reviewed the clinical recommendation and the routine application of mNGS approach in this context. To compile the existing knowledge about the direct investigation of non-surgical chronic wounds by mNGS, we conducted a literature review for studies applying shotgun and 16 S rDNA mNGS to chronic wound swabs and tissue biopsies for microbial screening.

## Methods

### Literature search

**Fig. 1:**
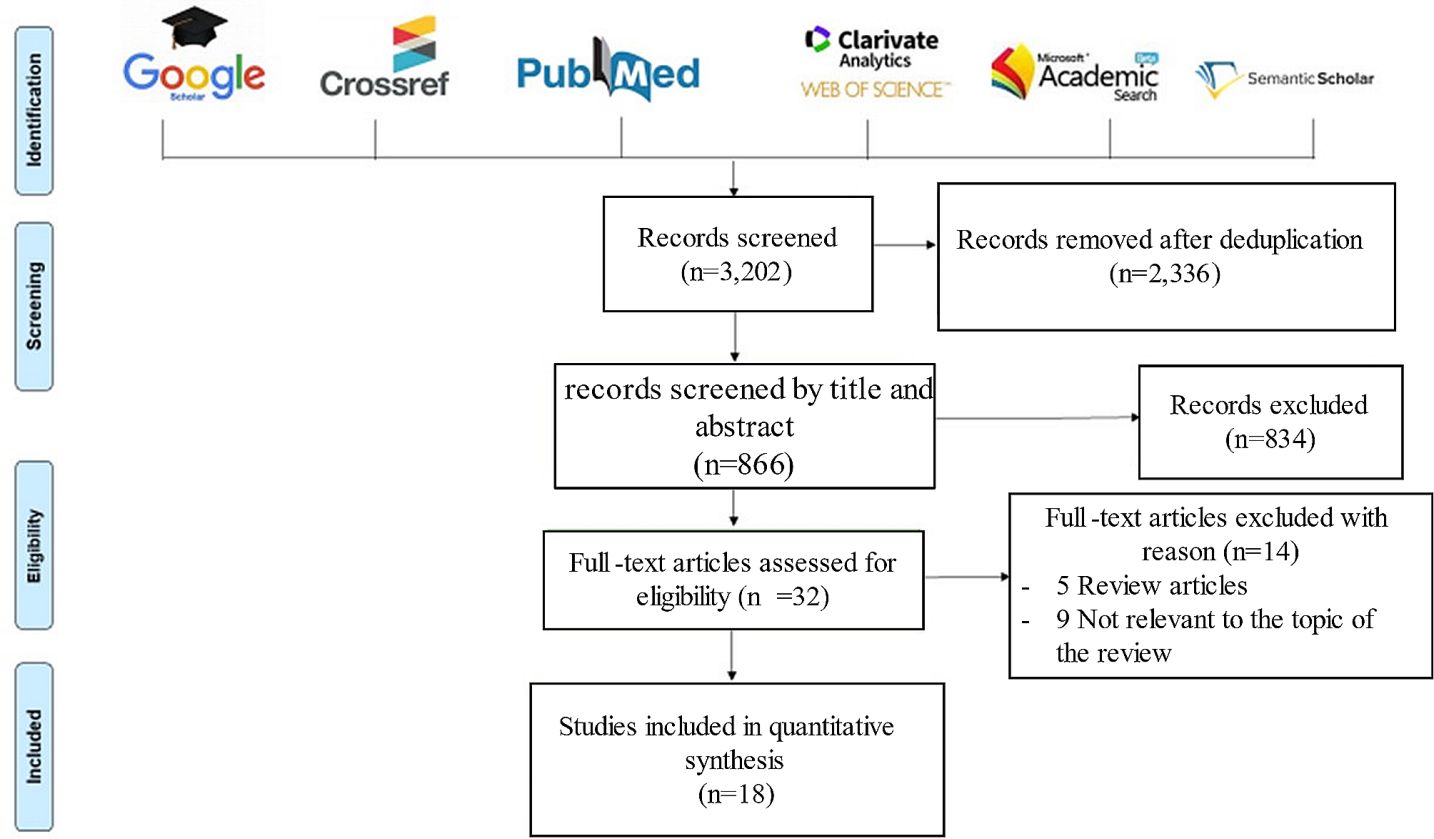
Literature search and study inclusion according to the PRISMA flow-chart. Six bibliographic databases were reviewed using the following keywords: “chronic wounds”, “chronic injuries”, “diabetic foot ulcer (DFU)”, “diabetes foot related diseases (DFRD)”, “diabetes”, “diab”, “decubitus ulcer (DU)”, “pressure ulcer (PU)”, “pressure injuries (PI)”, “venous leg ulcer (VLU)”, “diabetic foot infection (DFI)”, diabetic foot osteomyelitis (DFOM)”, “metagenomics”, “16S rRNA”, “shotgun”, “mNGS”, “microbiota”, “microbiome”, and “next generation sequencing (NGS)”, used alone and/or in combination, interested only to the mNGS application of CW samples

The literature search was conducted on PubMed, Google Scholar, Web of Science, Microsoft Academic, Crossref and Semantic Scholar databases according to the Preferred Reporting Items for Systematic Reviews and Meta-Analyses (PRISMA) guidelines [29]. We included studies published in English up until October 31, 2022, that were related to chronic wounds and metagenomic investigation. Duplicates were removed, and the remaining studies were screened by title and abstract according to the eligibility criteria. After reading the full text, only papers that met the eligibility criteria were selected for this review, using the following keywords: “chronic wound”, “chronic ulcer”, “chronic injuries”, “diabetic foot ulcer (DFU)”, “Diab”, “diabetes”, “diabetes foot related diseases (DFRD)”, “pressure ulcer (PU)”, “pressure injuries (PI)”, “decubitus ulcer”, “venous leg ulcer (VLU)”, “diabetic foot infection (DFI)”, diabetic foot osteomyelitis (DFOM)”, “metagenomics”, “16S rRNA”, “shotgun”, “mNGS”, “microbiota”, “microbiome”, and “next generation sequencing (NGS)”. These keywords were used in combination to perform an exhaustive search as presented in Fig. 1.

### Screening and inclusion

Studies that met the following criteria were included in this review: Studies applying mNGS on chronic wounds for: (1) case report; (2) prospective series; (3) retrospective series; (4) 16 S rRNA metagenomics; (5) Shotgun metagenomics application for either microbiome or diagnosis. Review articles, studies performed on animals, in vitro model optimisation, benchmarking, treatment optimisation studies, and non-clinical studies were excluded. Data extracted from the selected studies included first author’s name, year of publication, country, nucleic acid extraction method (including the commercial kit used), the sequencing platform, the type of mNGS (including the sequencing instrument, pipeline data analysis and software), reference microbial database, and the identified microorganisms. We included the total number of data yielded in both metagenomic approaches and data obtained by conventional in vitro culture. The data were extracted, cleaned, and selected by MM and FS and reviewed by CM and CDR, and then validated by AS and JPL.

The microbial colonisation of chronic wounds is notably diverse due to body and/or environmental microbiota translocations. To simulate the origin of the bacteria colonising the wounds and to understand the wound microbial colonisation dynamics, supplementary studies investigating the healthy gut, skin, urine microbiota, as well as environmental microbiome, were analysed for microbial comparison (Supplementary Figures S1, S2).

## Results

### Study selection

The database search identified 3,202 articles; 2,336 articles were removed after deduplication. Based on title and abstract screening, 834 articles were excluded. Of the 32 remaining articles, 14 were removed after full-text reading, including five review articles and nine articles that were not relevant to the topic of the review. Finally, 18 articles were included (Fig. 1).

### Studies characteristics

The first analysis of chronic wound microbiota by metagenomics was published in 2009, analysing 2,987 patients by 16 S rDNA pyrosequencing. This first and largest series included 916 VLUs, 910 DFUs, 791 PI, and 370 non-healing surgical wounds, identifying *Staphylococcus* spp. and *Pseudomonas* spp. as the most frequent species in 63% and 25% of all wounds, respectively [16]. Five studies were published between 2009 and 2018. Two studies were published in 2019 [30, 31], and 11 between 2020 and September 2022. Regarding the geographical origin of publications, nine studies were from Asia, including two publications each from Chinese, Indian, and South Korean laboratories [31–36]; one each from Saudi Arabia, Israel, and Taiwan [37–39]; four in the United States [16, 27, 40, 41]; four in Australia [30, 42–44] and only one from Europe, specifically France [45] (Supplementary Figure S1).

### Workflow

Among the 18 studies, 13 (72.22%) applied the metagenomic 16 S rDNA targeted protocol directly on clinical samples including swabs, tissue or bone biopsies (Table 1). Four studies used shotgun metagenomics on DFU biopsies, and only one study applied both shotgun and 16s rDNA approaches on DFI biopsies [38].

**Table 1:** Main characteristics of the studies included in this review. NI, No information

Reference	Country	Category	Sample type	Total samples	mNGS procedure	Sample preparation	sample preparation and DNA extraction	Sample enrichment	Targetted region	Library preparation	Sequencing Platform	Data analysis	Database
(Wolcott et al., 2009)	USA	Prospective	Ulcer debris	40	16s metagenomics	centrifugation at 14,000 rpm for 30 s and resuspended in 500 µl RLT buffer (Qiagen), 5 mm steel bead (Qiagen), 500 µl sterile 0.1 mm glass beads, TissueLyser (Qiagen), and run at 30 Hz for 5 min.	QIAamp DNA Mini Kit (Qiagen)	No		tag-encoded FLX and Titanium amplicon pyrosequencing (Roche)	Roche/454 GS FLX Titanium platform	NET and C# analysis pipeline with BLASTn	NCBI Bacterial database.
(Wolcott et al., 2016)	USA	Retrospective	Sharp debridement	2963	16s metagenomics	TissueLyser (Qiagen)	NI	No	V6, V3	HotStar- Taq master mix (Qiagen), Roche 454 protocols	Roche 454 platform	USEARCH UPARSE OTU selection algorithm	16s Classified sequences
(Gardiner et al., 2017)	Australia	Prospective	Swab	8	16s metagenomics	bead beating tube	BioStic DNA extraction kit (MO BIO Laboratories, Carlsbad, CA, USA)	No	V4	NexteraXT DNA Library Preparation Kit (Illumina)	Illumina Miseq	USEARCH v 1.8.1, QIIME, UCLUST method	Greengenes,
(Malone et al., 2017)	Australia	Retrospective	Tissue specimens	39	16s metagenomics	No	MoBio Power Biofilm DNA isolation kit (Mo Bio Cat)	No	V4	Q5 Hot Start High-Fidelity protocol and Nextera XT Index Kit (Illumina)	Illimina Miseq	CLC genomics workbench version 8.5.1	SILVA,
(Suryaletha et al., 2018)	India	Retrospective	Swab	100	16s metagenomics	No	Wizard Genomic DNA Purification kit (Promega, Madison, Wisconsin)	No	V3	Phusion Hot Start DNA Polymerase (Biolabs), Nextera XT Index Kit (Illumina)	Miseq illumina	PyNAST, RDP classifier	Greengenes
(Park et al., 2019)	South Korea	Retrospective	Biopsy and skin swab	20	16s metagenomics	No	RNeasy PowerMicrobiome kit (Qiagen, Hilden, Germany)	DNeasy PowerClean Pro clean-up kit (Qiagen),	V1-V3	Nextera XT index kit (Illumina)	Illumina Miseq	CLC genomic workbench	EzTaxon-e database
(Johani et al., 2019)	Australia	Prospective	Bone biopsy	20	16s metagenomics	No	DNeasy PowerBiofilm Kit (Qiagen, Hilden, Germany)	No	V3-V4	Nextera XT 384 index kit (Illumina)	Illumina Miseq	UCLUST	SILVA version 128
(Zou et al., 2020)	China	Prospective	Bone biopsy	28	16s metagenomics	No	DNA extraction kit (YiRui, ShenZhen, China)	No	V3-V4	PE250 sequencing protocol (Illumina)	Miseq illumina	MicroPITA software	Ribosome Database Project database
(Jnana et al., 2020)	India	Prospective	Swab	122	16s metagenomics	No	phenol-chloroform protocol	No	V2, V3, V4, V67, V8, and V9	Ion Xpress Barcode Adapters (Ion Torrent)	Ion Torrent	QIIME, MicroSEQ 16 S Reference library v2013.1, IonReporter software (v5.2)	Greengenes v13.5; BacDive database
(Moon et al., 2021)	South Korea	Prospective	Bone and soft tissues	54	16s metagenomics	No	PureLink Genomic DNA Mini Kit (Invitrogen, Carls- bad, CA)	No	full length of 16 S	Rapid Barcoding Sequencing Kit (SQK-RBK004; ONT)	Oxford Nanopore MinION	Metrichor/EPI2ME platform	NCBI 16 S bacterial database
(Saeb et al., 2021)	Saudi Arabia	Retrospective	Swab	38	16s metagenomics	One hour incubation at 37 °C with shaking, and TissueLyser (Qiagen)	Maxwell® 16 Cell DNA kits, Promega, Madison, WI, USA	No	V2-4-8 and V3-6, 7–9	16 S Ion Metagenomics Kit ™ (Thermo Fisher Scientific, Waltham, MA)	Ion PGM (Thermo Fisher Scientific)	Ion Reporter Software	Greengenes, MicroSEQ ID 16 S rRNA
(Kalan et al., 2021)	USA	Prospective	Levine’s swab	100	Shotgun metagenomics	No	PureLink Genomic DNA Mini Kit (Invitrogen)	NEBNext Microbiome DNA Enrichment kit (New England Biolabs)	No target	NexteraXT Library Preparation Kit (Illumina)	HiSeq 4000	in-house K-mer based algorithm refined, CosmosID, SUPERFOCUS software,	In-house microbial database,
(Chen et al., 2021)	China	Retrospective	DFU tissue	8	Shotgun metagenomics	No	DNeasy Blood and Tissue Kit (Qiagen, 69,504, Shenzhen, China)	No	No target	MGIEasy (MGIEasy universal DNA library prep kit)	BGISEQ platform	No	No
(Choi et al., 2021)	USA	Prospective	DFU biopsy	30	16s metagenomics	NI	NI	NI	NI	Ion Torrent platform	Ion Torrent platform	USearch7	GenBank database
(Radzieta et al., 2021)	Australia	Prospective	DFU biopsy	26	Shotgun metagenomics	TissueRuptor II homogoniser (Qiagen) and vortex for 10 s.	Zymo host zero microbial DNA kit (Zymo Research)	No	No target	Illumina Nextera DNA Flex Kit (Illumina)	HiSeq 2500	Humann2 pipeline,	ChocoPhlAn database (NCBI)
Dunyach-Remy et al., 2021	France	Prospective	deep tissue biopsy	24	16 S metagenomics	proteinase K at 56 °C for 3 h + MagNA Lyser (60s)	EZ1 DNA Tissue kit (Qiagen)	No	V3-V4 region	Illumina Nextera V2	Illumina Miseq	Uclust v1.2.22q, V2.2 method of RDP	Greengenes version 13 − 8
(Mudrik-Zohar et al., 2022)	Israel	Prospective	Biopsy	31	16s metagenomics and shotgun metagenomic	No	DNeasy PowerBiofilm Kit (Qiagen)	NEBNext Microbiome DNA Enrichment Kit (New-England Biolabs, USA)	V4	Nextera XT Index Kit (Illumina)	Illumina MiniSeq, Illumina NextSeq 500	QIIME2	SILVA
(Yang et al., 2022)	Taiwan	Prospective	Tissue sample	1	Shotgun metagenomics	Grind and vortex for 30 min at 3000 rpm with 1 g of 0.5 mm glass beads.	DNeasy Blood and Tissue Kit (Qiagen)	End-repaired adapter and polymerase chain reaction amplification	No target	MGIEasy FS DNA Library Prep Kit (MGI)	DNBSeq-G50 platform	BWA	NCBI microbial reference genomes (RefSeq)

### DNA extraction

Depending on the sample origin, genomic DNA was extracted following specific protocols. Mechanical and enzymatic pretreatments were needed to increase the DNA extraction from swabs, tissue, and bone biopsies of chronic wounds. Chemical treatment by Tissue Lyser solution (Qiagen, Hilden, Germany) was used with or without prior incubation at 37 °C in five studies, followed by a vortexing step to destroy bacterial cells. A supplementary incubation with proteinase K at 56 °C was recommended before DNA isolation [16, 27, 37, 42, 45]. In specific protocols, the mechanical treatment using steel and glass beads was combined with enzymatic and chemical procedures directly applied on chronic wounds tissue or bone biopsies performed after debridement and followed by manual or automatic DNA extraction (Table 1). Further post-extraction treatments, including microbial genome enrichment using bead-based captor, nonspecific amplification, and host genome removal, were applied prior to library preparation to improve the microbial genome detection [31, 38, 39, 41].

### Metagenomic analysis

**Fig. 2:**
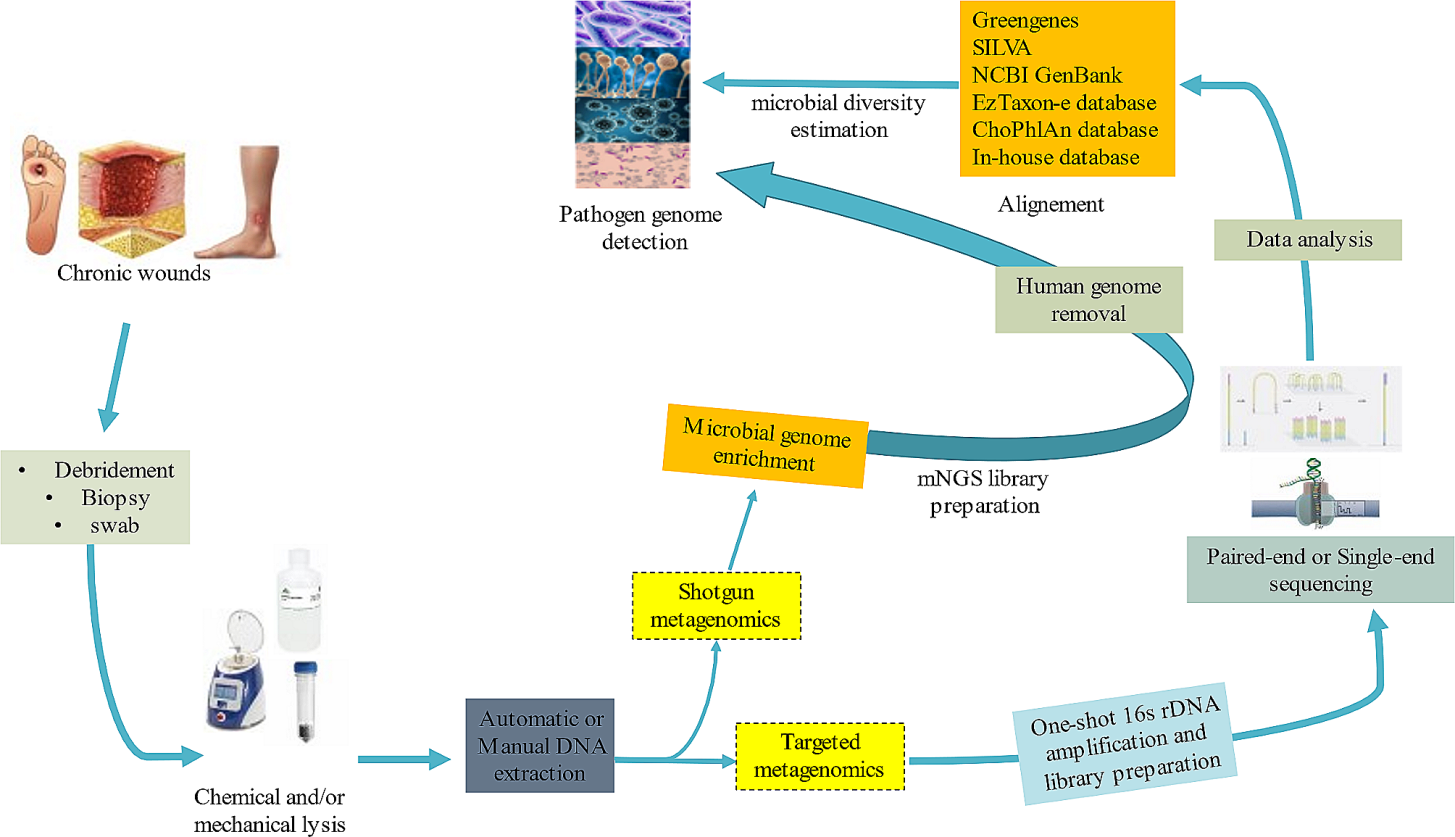
Metagenomic workflow applied to chronic wound samples. Clinical swabs and biopsies underwent chemical and mechanical lysis, after which microbial DNA was extracted using either a manual or automatic protocol. Post-DNA extraction treatment was performed for microbial genome enrichment and human genome removal. The remaining DNA was used for single-end or paired-end library preparation, following either shotgun or 16 S rDNA targeted mNGS protocols, and then sequenced according to the sequencing platform. Microbial genomes were identified by alignment against local or online databases using either in-house or commercial pipelines

In order to estimate the microbial diversity based on DNA analysis, targeted metagenomics was applied directly on clinical samples in 13/18 (76.5%) studies, amplifying the full or partial 16 S rRNA encoding gene following an in-house or commercially developed PCR (Table 1; Fig. 2).

The V3 and V4 hypervariable rDNA regions were targeted in 10/13 studies, using one-shot amplification and library preparation procedure (Supplementary Table 1). Unique amplification of V4 or V3 regions was noted in three [38, 43, 44] and one [35] studies, respectively. In the five remaining studies, a double amplification was applied targeting V3-V4 or V1-V3 or V3-V6 16 S rDNA variable regions [30, 31, 38, 39, 41].

A DNA library was constructed using 16 S rDNA amplification following the Illumina Nextera-XT paired-end sequencing protocol (Illumina, San Diego, USA). An Ion-Torrent commercial multiplex amplification targeting most variable 16 S rDNA regions V2, V3, V4, V6, V7, V8, and V9 was used in two studies, followed by Ion Xpress Barcode Adapters library preparation protocol and Ion-Torrent sequencing [34, 37]. Full length 16 S rDNA gene was sequenced in only one study using Rapid Barcoding Sequencing Kit (Oxford Nanopore technologies, Oxford Science Park, UK) [33]. For global and real microbiome detection in chronic wound samples at species-level identification, shotgun metagenomics was applied with no prior amplification and no specific target (Table 1).

Microbial genome enrichment increases the possibility of microbial genome detection. Two different approaches were used, either by human genome depletion using NEBNext Microbiome DNA Enrichment kit (New England Biolabs, Ipswich, USA) [38, 41], or with non-specific random amplification after end-repaired adapters [39], followed by paired-end deep sequencing.

### Data analysis

For 16 S rDNA or shotgun metagenomic investigation, commercial or in-house developed pipelines were used for data analysis according to the sequencing procedure (Table 1). Exhaustive analysis of shotgun metagenomes usually started with human genome removal by alignment of total reads against a reference human genome using WBA software [39], or with HUMAnN2 pipeline The filtered reads were again aligned against the NCBI GenBank database or a specific in-house microbial database constructed from GenBank, using an adaptive algorithm [36, 39, 41, 42]. Bacterial diversity based on targeted metagenomics was estimated by aligning the 16 S data using an adaptive pipeline against a specific database (Fig. 2). Greengenes, SILVA, and NCBI GenBank were the most commonly used reference databases for microbiome analysis (Table 1, Supplementary Figure S1).

### Chronic wound microbiology

**Fig. 3:**
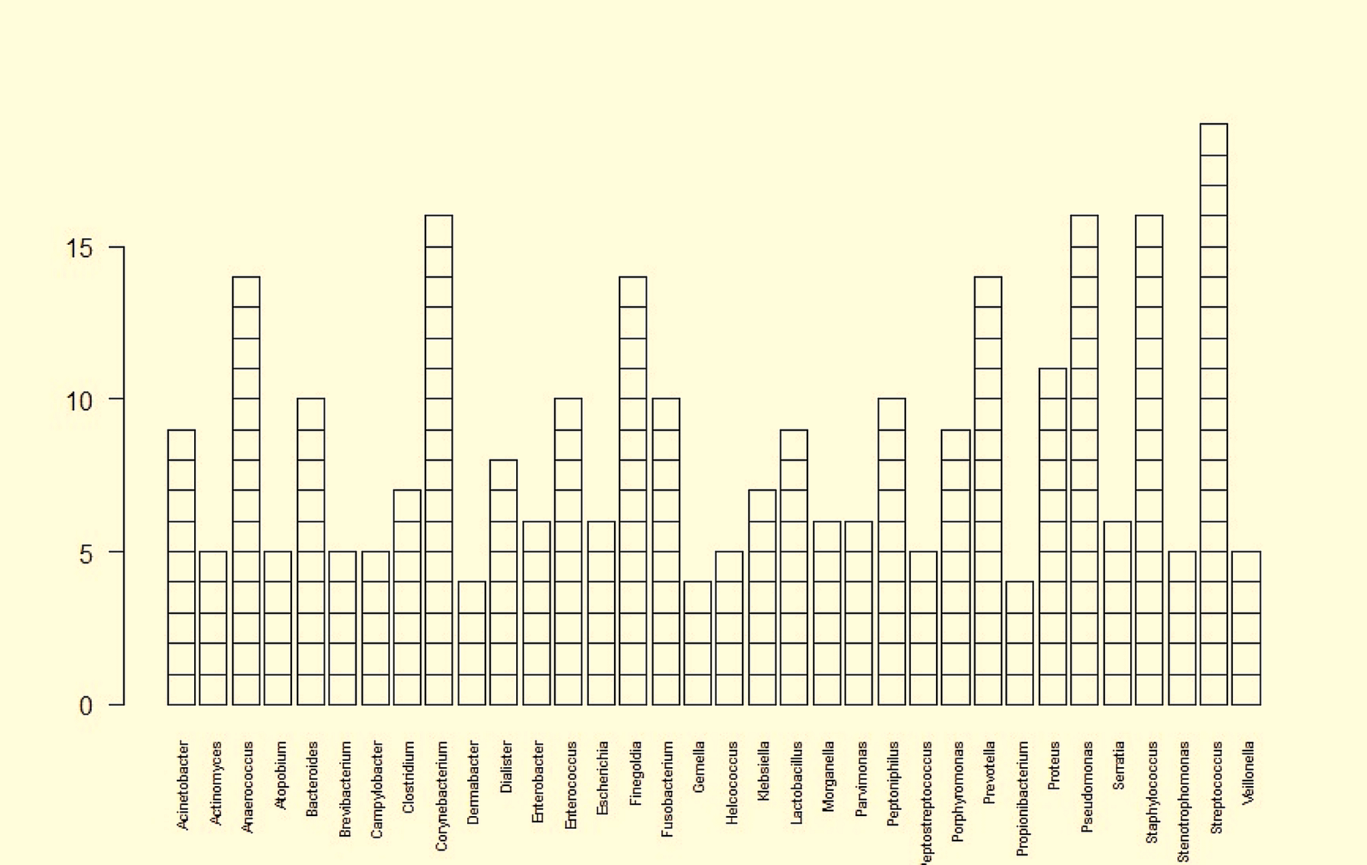
Most abundant bacterial genera detected by mNGS in chronic wounds. Out of a total of 160 bacterial genera detected in all chronic wounds, *Streptococcus, Pseudomonas, Corynebacterium, Finegoldia, Dialister, Anaerococcus, Prevotella*, and *Bacteroides* were the most common bacteria colonising the chronic wounds and detected in these lesions. *Anaerococcus, Bacteroides, Corynebacterium, Finegoldia, Fusobacterium, Peptinophilus, Prevotella, Pseudomonas, Staphylococcus*, and *Streptococcus* were reported in more than 75% of studies. The prevalence of the remained genera depended on the nature of the chronic wounds and the colonisation/infection stage

Wound evolution could be defined by microbial diversity and the colonising bacteria, which may progress into an infection [46, 47]. More than 400 bacterial species were isolated from different chronic wounds [48], but more than 1,000 bacteria colonising human skin could generate the wound microbiome, on tributing to wound evolution [49, 50]. Focusing only on the most abundant bacteria in chronic wounds, 164 bacterial genera were identified by both 16 S and shotgun metagenomics. *Streptococcus* was the most common bacteria genus, followed by *Staphylococcus, Pseudomonas, Corynebacterium*, and some anaerobes (*Prevotella, Finegoldia, Anaerococcus*) (Fig. 3; Table 2).

**Table 2:** Microbiology of chronic wounds obtained by metagenomics and included in this review. DFI, diabetic foot infection; DFOM, diabetic foot osteomyelitis; DFU, diabetic foot ulcer; PU, pressure ulcer; VLU, venous leg ulcer

References	Sample type	Number	Wound	Detected genera
(Wolcott et al., 2009)	Ulcer debris	40	VLU	*Actinomyces, Alistipes, Anaerococcus, Arthrobacter, Bacteroides, Brevibacterium, Campylobacter, Candidatus Amoebinatus, Corynebacterium, Desulfovibrio, Dialister, Enhydrobacter, Enterobacter, Escherichia, Fastidiosipila, Finegoldia, Fusobacterium, Gallicola, Gemmatimonas, Granulicatella, Helcococcus, Lactobacillus, Morganella, Nocardioides, Oligella, Peptoniphilus, Peptostreptococcus, Petrimonas, Porphyromonas, Prevotella, Propionibacterium, Proteus, Pseudomonas, Riemerella, Serratia, Sphingobium, Sporobacter, Staphylococcus, Streptococcus, Terrimonas, Xylella*
(Wolcott et al., 2016)	Sharp debridement	2,963	DFU, VLU, PU	*Acinetobacter, Anaerococcus, Bacteroides, Corynebacterium, Delftia, Enterobacter, Enterococcus, Finegoldia, Flavobacterium, Fusobacterium, Peptoniphilus, Prevotella, Propionibacterium, Proteus, Pseudomonas, Serratia, Stenotrophomonas, Streptococcus, Staphylococcus*
(Gardiner et al., 2017)	Swab	8	DFU	*Actinomyces, Bacteroides, Brevibacterium, Clostridium, Corynebacterium, Deinococcus, Devosia, Dietzia, Fusobacterium, Jeotgalicoccus, Lactobacillus, Megamonas, Methylobacterium, Methylopila, Neisseria, Paracoccus, Phascolarctobacterium, Phenylobacterium, Porphyromonas, Prevotella, Rheinheimera, Rubellimicrobium, Rubrobacter, Sphingobacterium, Sphingomonas, Spirosoma, Staphylococcus, Streptococcus, Sutterella, Wolbachia, Ruminococcus*
(Malone et al., 2017)	Tissue specimens	39	DFI	*Acinetobacter, Anaerococcus, Blastocatella, Corynebacterium, Enterobacter, Finegoldia, Haemophilus Peptoniphilus, Porphymonas, Prevotella, Proteus, Pseudomonas, Staphylococcus, Streptococcus*
(Suryaletha et al., 2018)	Swab	100	DFU	*Aclanivorax, Alcaligenes, Anaerococcus, Bacteroides, Balneimonas, Candidatus Flomobacter, Candidatus solibacter, Cetobacterium, Clostridium, Corynebacterium, Desulfococcus, Dialister, Esherichia, Facklamia, Facklamia, Filifactor, Finegoldia, Granulicatella, Helcococcus, Ignatzschineria, Klebsiella, Mogibacterium, Morganella, Moryella, Mycoplasma, Myroides, Oribacterium, Paracoccus, Parvimonas, Peptococcus, Peptostreptococcus, Photobacterium, Prevotella, Pseudomonas, Stenotrophomenas, Streptococcus, Trabulsiella, Vagococcus, Veillonella, Veillonella*
(Park et al., 2019)	Biopsy and skin swab	20	DFU	*Anaerococcus, Bacteroidetes, Dialister, Lactobacillus, Finegoldia, Peptoniphilus, Porphyromonas, Prevotella, Streptococcus*,
(Johani et al., 2019)	Bone biopsy	20	DFI	*Achromobacter, Acinetobacter, Actinobaculum, Actinomyces, Anaerococcus, Arcanobacterium, Atopobium, Bacillus, Bacteroides, Bifidobacterium, Brevibacterium, Bulleidia, Campylobacter, Clostridium, Corynebacterium, Deinococcus, Dermabacter, Dialister, Eikenella, Enterococcus, Facklamia, Faecalibacterium, Finegoldia, Fusobacterium, Gallicola, Gemella, Helcococcus, Lactobacillus, Micrococcus, Morganella, Moryella, Parvimonas, Pasteurella, Peptococcus, Peptoniphilus, Peptostreptococcus, Planctomyces, Porphyromonas, Prevotella, Propionibacterium, Proteus, Providencia, Pseudomonas, Serratia, Slackia Sphingomonas, Staphylococcus, Stenotrophomonas, Streptococcus, Tannerella, Treponema, Varibaculum, Veillonella, Wohlfahrtiimonas*
(Zou et al., 2020)	Bone biopsy	28	DFOM	*Anaerococcus, Bacteroides, Bradyrhizobium, Citrobacter, Corynebacterium, Dialister, Enterococcus, Finegoldia, Fusobacterium, Halomonas, Klebsiella, Porphyromonas, Prevotella, Providencia, Pseudomonas, Staphylococcus, Streptococcus, Veillonella*
(Jnana et al., 2020)	Swab	122	DFU	*Achromobacter, Acinetobacter, Alcaligenes, Bacillus, Burkholderia, Corynebacterium, Methylobacterium, Pseudomonas, Staphylococcus, Streptococcus*
(Moon et al., 2021)	Bone and soft tissues	54	DFI	*Achromobacter, Aeromonas, Anaerococcus, Bacteroides, Corynebacterium, Enterobacter, Enterococcus, Escherichia, Finegoldia, Fusobacterium, Klebsiella, Lactobacillus, Morganella, Parvimonas, Peptoniphilus, Proteus, Pseudomonas, Serratia, Staphylococcus, Streptococcus, Prevotella*
(Saeb et al., 2021)	Swab	38	DFU	*Acinetobacter, Actinomyces, Brevibacterium, Corynebacterium, Dermabacter, Enterococcus, Klebsiella, Proteus, Pseudomonas, Serratia, Staphylococcus, Streptococcus*
(Kalan et al., 2021)	Levine’s swab	100	DFU	*Corynebacterium, Pseudomonas, Staphylococcus, Streptococcus*
(Chen et al., 2021)	DFU tissue	8	DFI	*Aerococcus, Alloprevotella, Alterileibacterium, Anaerococcus, Atopobium, Bacteroides, Bulleidia, Campylobacter, Clostridium, Coprobacter, Criibacterium, Eggerthia, Erysipelotrichaceae, Eubacterium, Ezakiella, Facklamia, Fenollaria, Filifactor, Finegoldia, Fusobacterium, Gemella, Gordonibacter, Hallella, Helcococcus, Kallipyga, Klebsiella, Lachnospiraceae, Lagierella, Levyella, Mageeibacillus, Massiliomicrobiota, Mogibacterium, Neofamilia, Odoribacter, Olegusella, Peptoanaerobacter, Peptoniphilus, Peptostreptococcus, Phocaeicola, Porphyromonas, Prevotella, Shuttleworthia, Solobacterium, Streptococcus, Tannerella, Tissierellia, Urinacoccus, Varibaculum, Veillonellaceae*
(Choi et al., 2021)	DFU biopsy	30	DFI	*Anaerococcus, Atopobium, Corynebacterium, Enterococcus, Escherichia, Finegoldia, Gemella, Klebsiella, Lactobacillus, Morganella, Parvimonas, Peptoniphilus, Prevotella, Proteus, Staphylococcus, Streptococcus*
(Radzieta et al., 2021)	Tissue biopsy	26	DFU	*Achromobacter, Acinetobacter, Actinobaculum, Actinomyces, Alloprevotella, Anaerococcus, Arcanobacterium, Atopobium, Bacteroides, Bifidobacterium, Brevibacterium, Brevundimonas, Campylobacter, Citrobacter, Clostridiales, Clostridium, Comamonas, Coprobacillus, Corynebacterium, Dermabacter, Dialister, Dietzia, Dolosigranulum, Eggerthella, Eikenella, Enhydrobacter, Enterococcus, Escherichia, Finegoldia, Fusobacterium, Gemella, Granulicatella, Haemophilus, Helcococcus, Lactobacillus, Massilia, Morganella, Murine, Nocardioides, Parvimonas, Pasteurella, Peptoniphilus, Peptostreptococcus, Porcine, Porphyromonas, Prevotella, Propionibacterium, Propionimicrobium, Proteus, Providencia, Pseudomonas, Rhodopseudomonas, Solobacterium, Staphylococcus, Streptococcus, Tannerella, Varibaculum, Veillonella*
(Dunyach-Remy et al., 2021)	Deep tissue biopsy	24	PU	*Acidovorax, Acinetobacter, Anaerococcus, Atopobium, Clostridium, Corynebacterium, Dermabacter, Dialister, Enterococcus, Escherichia, Finegoldia, Fusobacterium, Klebsiella, Lactobacillus, Parvimonas, Peptococcus, Peptoniphilus, Porphyromonas, Proteus, Pseudomonas, Sphingomonas, Sporobacterium, Staphylococcus, Streptococcus, Tepidimonas*
(Mudrik-Zohar et al., 2022)	Biopsy	31	DFI	*Bacteroides, Bifidobacterium, Campylobacter, Clostridium, Dialister, Eggerthella, Eikenella, Flavonifractor, Fusobacterium, Intestinimonas, Lachnoclostridium, Lactobacillus, Mobiluncus, Moraxella, Ornithobacterium, Parabacteroides, Peptoniphilus, Prevotella, Proteus, Pseudomonas, Roseburia, Streptococcus, Veillonella*
(Yang et al., 2022)	Tissue sample	1	DFU	*Candida, Pseudomonas, Staphylococcus*

**Fig. 4:**
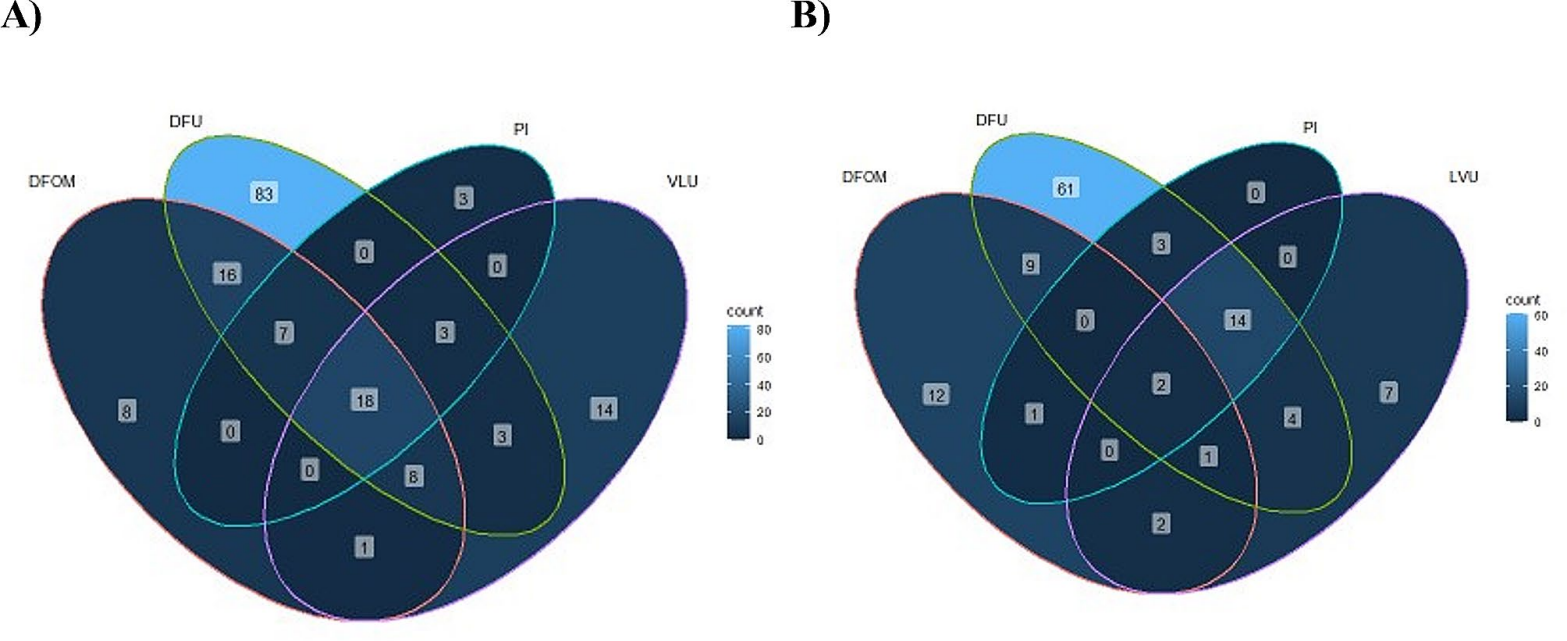
Venn diagrams illustrating the distribution of microorganisms identified by metagenomic in the different chronic wounds (DFU, diabetic foot ulcer; PI pressure injuries; VLU, venous leg ulcer; DFOM, diabetic foot osteomyelitis). **A**) Venn diagram of the 164 genera identified by mNGS across all included studies. DFUs are the most diversified wounds with 138 genera, followed by VLUs with 41 genera, PIs with 30, and DFOMs with 27. **B**) Venn diagram of the 116 microbial species identified by both 16s and shotgun metagenomics across 13 studies. A total of 94 species were identified in DFU samples, followed by VLU with 30 species, DFOMs with 27, and PIs with only 20 species

*Pseudomonas, Corynebacterium*, and anaerobic bacteria such as *Finegoldia, Dialister, Anaerococcus, Prevotella*, and *Bacteroides* were the most common bacteria colonising the chronic wounds and were detected in all types of lesions. Moreover, aerobic Gram-positive cocci, *Staphylococcus* and *Enterococcus*, were detected in both DFRDs (DFU and DFOM) and VLU. Aerobic Gram-negative bacilli belonging to Enterobacteriaceae were isolated from DFU (*Morganella, Providencia* and *Citrobacter*), DFOM (*Providencia* and *Citrobacter*), and VLU (*Morganella*), whereas other Gram-negative bacilli were detected in DFU (*Sphingomonas, Xylella and Tepidimonas*), PU (*Sphingomonas, and Tepidimonas*), and VLU (*Xylella*). Interestingly, anaerobes were particularly present. Anaerobic Gram-negative bacilli were isolated from DFU and DFOM (*Veillonella, Fusobacterium, Porphyromonas, Enhydrobacter* and *Terrimonas*) and VLU (*Fusobacterium, Porphyromonas, Enhydrobacter* and *Terrimonas*). Moreover, anaerobic Gram-positive cocci were detected in DFU (*Parvimonas, Peptostreptococcus*, and *Peptococcus*), VLU (*Peptostreptococcus*) and PI (*Parvimonas*, and *Peptococcus*). Anaerobic Gram-positive bacilli were detected in chronic wounds included: DFU (*Clostridium, Brevibacterium, Actinomyces*, and *Atopobium*), VLU (*Brevibacterium* and *Actinomyces*) and PI (*Clostridium* and *Atopobium*). Finally, some fastidious bacteria such as *Granulicatella, Helcococcus Campylobacter, and Nocardioides* were detected in DFU and VLU, whereas *Dermabacter* was detected in DFU and PI (Fig. 4; Table 2).

DFRDs were the wounds with highest microbial richness, with a total of 147/157 bacterial genera including 107 genera detected only in DFU and DFOM samples, compared to 47 from VLU, including 14 detected only in VLU samples. In contrast, PI is the least colonised wound, with a total of 31 bacteria genera identified (Fig. 4). Supplementary information about fungal (*Aspergillus* and *Candida*) and viral species was provided by shotgun metagenomics, as well as identification of *Staphylococcus* and *Pseudomonas* bacteriophages that could be used as a therapeutic approach to treat chronic wound infection [27, 39, 41].

A deep 16 S rDNA and shotgun-based metagenomics investigation yielded 116 microbial species in the 13 selected studies. A total of 100 species were detected in DFRD samples, including 96 species in DFU with 66 in DFU only, 10 in DFOM biopsies with five in DFOM only, 30 in VLU with nine in VLU only, 20 species only detected in PIs. These species were mostly represented by *S. aureus, S. agalactiae, Escherichia coli, Pseudomonas aeruginosa, Corynebacterium striatum, Corynebacterium tuberculostearicum*, and some anaerobes (*Finegoldia magna, Peptoniphilus harei, Anaerococcus vaginalis*, and *Prevotella bivia*) (Fig. 4, Supplementary Table S2). Based on wound colonisation, 16 bacteria species were shared between DFRD, PI and VLU (*S. aureus, S. epidermidis, Staphylococcus haemolyticus, Staphylococcus lugdunensis S. agalactiae*, *E. faecalis, Enterobacter hormaechei, P. aeruginosa, Stenotrophomonas maltophilia, Acinetobacter baumannii, Delftia acidovorans, Corynebacterium jeikeium, C. striatum, C. tuberculostearicum, A. vaginalis*, and *F. magna*) [16]. *Staphylococcus pettenkoferi*, *E. coli, Serratia nematodiphila, Actinomyces europaeus*, and *P. harei* were specifically detected in DFU and PI. *Klebsiella pneumoniae, Prevotella denticola, Prevotella fusca*, and *Veillonella parvula* were identified from DFU and DFOM. *Proteus mirabilis*, *Fusobacterium nucleatum*, and *P. bivia* were detected in DFU and PI, and only *Bacteroides fragilis* were identified from DFOM and PI (Fig. 4).

With no prior target, shotgun metagenomics identified the presence of Epstein Barr Virus (EBV), involved in non-healing DFU through association with NK/T-cell-lymphoma [39]. In addition to bacterial and viral detection, shotgun mNGS detected the presence of *Candida albicans*, *Candida glabrata, Candida tropicalis* and *Aspergillus* spp. in DFU and VLU samples [23].

### Prediction of wound-colonising microbe ecology

To understand the process of chronic wound colonisation and the potential origin of the microorganisms colonising the wound, we compared the wound microbiome and other body microbiomes. Unfortunately, only two studies compared skin and wound microbiomes [31, 43]. To complete this analysis, we recovered healthy skin, urine, and gut microbiota present in the literature [51–56] (Supplementary Figure S2). At least 90 bacterial genera colonising the chronic wound could be translocated from the different body microbiota. Gut microbiota was the principal source of wound-colonisation microorganisms (53.5%), followed by cutaneous (17.2%) and urine (12.1%) microbiota (Supplementary Table S3). The remaining 67 microorganisms potentially belonged to other body microbiota and environmental microbiota, possibly transported by healthcare professionals and the hospital ecosystem.

Microbial diversity in chronic wounds according to geographical distribution was highest in Asian populations, with 31.2% of bacterial genera identified compared to 19.2% in Australians and 10.2% in Americans. The microbial diversity of wounds in European and Arabic and Middle-Eastern populations were the lowest, with only a mean of 3 and 8 microorganisms identified, respectively (Supplementary Figure S3). *Staphylococcus, Streptococcus, Enterococcus, Proteus, Pseudomonas, Acinetobacter, Corynebacterium, Lactobacillus, Dialister, Fusobacterium*, and *Peptoniphilus* were the most common bacteria genera identified in chronic wounds worldwide. *Escherichia* and different anaerobes (*Finegoldia, Anaerococcus, Parvimonas, Atopobium*, and *Porphyromonas*) were preferentially detected in chronic ulcers from American, Asian, Australian, and European patients, whereas *Campylobacter, Prevotella*, and *Bacteroides* were only identified in American, North African, Middle-Eastern, Asian, and Australian patients. Interestingly, *Klebsiella* was not detected in wounds from Australian patients and *Clostridium* in ulcers from American patients. Twenty-six bacterial genera were shared between American, Asian, and Australian patients in which *Morganella, Enterobacter*, and different fastidious (*Gemella, Helcococcus*, *Granulicatella*) or anaerobic bacteria (*Peptostreptococcus*) were only identified in these populations. The low microbial diversity in chronic wounds from European, North African, and Middle-Eastern patients could be due to the limited number of studies (*n* = 3) available in these populations (Table 1, Supplementary Figure S3).

### Comparison of microbiome between infected and non-infected chronic wounds

**Fig. 5:**
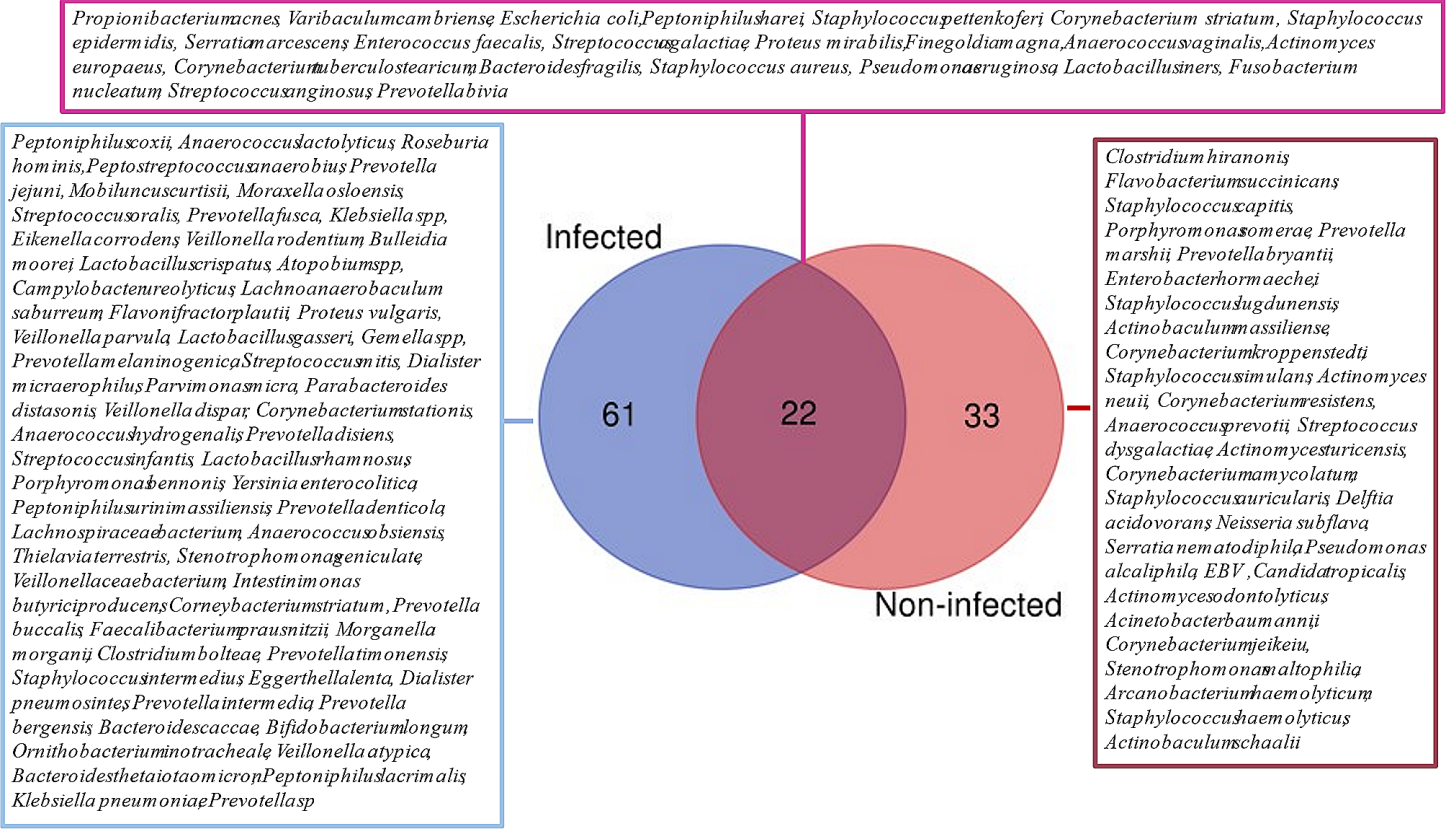
Venn diagram illustrating the distribution of microbial species between infected and non-infected chronic wounds. Out of the 116 microbial species identified in all selected studies, 61 species were only detected in infected wounds, 33 exclusively found in non-infected wounds, and 22 were present in both

Based on clinical evolution, chronic wounds were divided into two categories: (1) Infected wounds including DFI and DFOM; (2) Non-infected wounds including DFU, VLU, and PI. Microbial comparison between the two groups highlighted a high microbial diversity in infected wounds compared to non-infected ulcers. A total of 73 microbial species were detected in the infected wounds including a majority of anaerobic bacteria (63.6%) while only 54 species were detected in the non-infected category, in which Gram-positive bacteria were predominant (61.1%) (Fig. 5). Both categories shared 18 bacterial species, represented by common Gram-positive cocci (*S. aureus, S. agalactiae, S. pettenkoferi, E. faecalis, Streptococcus anginosus*), Gram-negative bacilli (*E. coli, P. mirabilis, P. aeruginosa*) and anaerobes (*P. harei, F. magna, (A) vaginalis*, *F. nucleatum, P. bivia, (B) fragilis* and *Varibaculum cambriense*). The majority of these species are pathogens and involved in infection and/or the worsening evolution of the chronic wounds.

### Limitations

There are three main limitations of the studies. Fistly, fungal infections are a major contributor to delayed wound healing, involved in polymicrobial biofilm formation and helping commensal bacteria in resisting antibiotics and the host immune response [57, 58]. However most studies (12/17) used 16 S rDNA metagenomics, which only detected bacteria, leaving wound viral, fungal and/or parasites communities underestimated and specially fungal infections undocumented [27, 39]. Secondly, the final inclusion of patients was restricted by the extracted DNA quantity and the sampling methods. Low DNA concentrations reduced the depth of sequencing, which limited further genomic and taxonomic investigations [45]. Swabs were mainly used for superficial wound sampling, although this technique should be avoided, due to its sensitivity, which restricts the identification of anaerobic bacteria. More in-depth sampling methods should be considered [34, 35, 37, 41, 43]. Finally, DNA extracted from wound swabs and biopsies generated a high human genome level, which required microbial genome enrichment and/or human genome depletion to improve the microbial genome detection [31, 38, 39, 41]. The low level of bacterial genera detected from DFOM and PI could be due to the complexity of DNA extraction and the human genome fraction [30, 32, 45]. More studies should investigate DNA extraction methods in combination with microbial genome enrichment to improve the detection of microorganisms in the sequenced samples [42, 59]. Alternatively, human DNA depletion prior to library preparation could remove some microbial genomes and genomic signatures of DNA viruses (such as EBV able to be integrated into the human genome [39]), leaving a part of the microbial documentation unexplored.

An additional limitation arose from the data analysis, focusing either on in-house pipelines or commercial software using old versions of reference databases, which could have misreported taxonomic classification of more recently described bacterial species. Regular updates of the reference microbial databases or amalgamation of several reference databases are needed for an exhaustive classification of the sequenced microorganisms (Table 1). Finally, the simulation of the wound-colonising microorganisms was based on healthy skin, urine, and gut microbiota from the literature, which was insufficient to clarify the source of this colonisation. A comparison of multiple body and wound microbiota as well as environmental microbiome is recommended to better understand the wound colonisation.

## Discussion

Delayed wound healing including non-surgical chronic wounds affects more than 100 million individuals worldwide and cost over $31 billion in patient care and treatment [13, 50]. Clinical outcomes of these lesions could be related to the origin of the microorganisms colonising the wound from diverse body microbiota and environmental contamination, involved in biofilm formation and infection [2, 13, 60, 61]. The difficulties in distinguishing between wound colonisation and infection represent one of the factors that delay treatment and wound healing [13, 41, 59, 62–64], faced with routine microbiology limited by selective bacteria cultures [2, 8, 65]. Shotgun and 16s mNGS allowed to investigate wound microbiota diversity, its origin and infection [31, 41, 43, 66]. This review of 18 original studies documented 164 bacteria genera detected by mNGS in chronic wounds and successfully identified 116 microbial species (Fig. 5), demonstrating a high variability of bacteria present in these wounds. In addition to traditional investigations looking strictly at bacterial communities, mNGS also detected fungi and viruses colonising these wounds. *Candida* and *Aspergillus* were the most detected fungi [27, 39], and once EBV was documented in a non-healing wound EBV-associated NK/T cell lymphoma [39]. Most of these non-bacterial microorganisms are missed by the current in vitro approaches and even several molecular tools.

Global comparison between microbiota of different chronic wounds showed that DFRD had the greatest diversity (more than 87% of the detected microorganisms) (Table 2), whereas VLU and PI had the lowest. This result could be due to the low number of articles studying VLU and PI microbiomes, as well as the difficulties encountered with sampling, storage, DNA extraction, and mNGS library preparation [45]. The high similarity between chronic wound microbiota and other body microbiota (Supplementary Fig. 3) is suggestive of translocation of body microbiota to the wound [43, 63, 66] (Fig. 4). Geographical location influences the body microbiota [67], and here was shown to affect the diversity of microorganisms colonising the wounds. The high diversity in Asian patients may be due to the patient’s physiology, cultural and individual habits, hygiene, lifestyle, socioeconomic factors, patient ecology, and climate [68]. However, it is important to note that, despite this geographical distinction in wound-colonising microorganisms, the main microorganisms present on chronic wounds and influencing their evolution remained the same worldwide including Gram-positive cocci (*Streptococcus, Staphylococcus, Enterococcus*) and bacilli (*Corynebacterium*), Gram-negative bacilli (*Pseudomonas, Acinetobacter, Proteus*) and an anaerobe (*Peptoniphilus*) [61]. Based on wound evolution, infected wounds had a greater microbial diversity than non-confirmed infected wounds (Fig. 5). Among these microorganisms, the identification of biofilm-forming bacteria in infected or non-infected wounds could provide additional information on the worsening evolution of wounds [69–71]. These bacteria are frequently associated with anaerobes, which interfere with the inflammatory response and remodel wound healing processes [72]. Moreover, the presence of certain pathogenic Gram-negative bacteria (*Escherichia*, *Klebsiella* and *Pseudomonas*) increases the worsening evolution of the wound due to the high secretion of virulence factors, their potential for immune evasion, and their antiphagocytosis activity [73]. Non-fungal investigation is the most limit encountered here, despite their high contribution in chronic wound healing delay, only shotgun investigation had been added supplementary information about fungi infecting wounds [27, 39], which leaves part of the microbiome in obscurity.

A key point in the management of chronic wounds is the importance of multidrug resistance. Interestingly, information about antiseptic and antibiotic resistance could be predicted *in silico* by shotgun mNGS [37, 45, 59], as well as pathogen genotypes determined by microbial genome analysis [33, 34, 40]. In the future, continuous surveillance of wound evolution according to the microbial colonisation throughout treatment could help clinicians manage the wounds by revealing the role of bacteria in wound healing and patient outcomes [1, 60, 63, 74, 75].

New therapeutic approaches can be applied based on metagenomic results. According to an experimental study, the dominance of probiotic bacteria like *Lactobacillus* and *Bifidobacterium* could promote the healthy microbiome by controlling the wound colonisation that leads to wound healing [62, 76, 77]. Moreover, shotgun mNGS may add supplementary information about bacteriophages, which could be used as an alternative for phage therapy against biofilm-forming bacteria such as *Staphylococcus* species [41, 59, 60], as demonstrated by in vitro investigations of lytic activity of the Rosa-like phage against *S. aureus*, providing a phage therapy treatment for DFRD [78]. Unfortunately, despite the advantage offered by mNGS, this technology has been little used in chronic wounds microbiome investigation, while RNA and DNA viruses were also poorly investigated, obscuring a part of the wound microbiology. This may be due to the difficulties encountered in DNA extraction from the clinical samples usually studied by swabs and biopsies and the sample storage [43], which requires more optimisation of DNA extraction and sequencing protocols.

With the emergence of real-time sequencing, bacteria detection and profiling could be performed within hours of the patient’s admission, which will improve the management of the patients and reduce the risk of wound complications [69, 70]. Molecular methods should be adopted in routine microbiology to identify microbes escaping conventional cultures. These cost and time-effective innovative technologies are promising tools to better understand the local ecology of chronic wounds, to help clinicians to differentiate colonisation more accurately from infection, and to optimise an adaptive treatment based on wound microbiome. However, some difficulties will have to be overcome. The sensitivity of molecular techniques can detect non-viable microorganisms disturbing the data interpretation. Moreover, it would be necessary to categorize all bacteria (genus or species) identified by bioinformaticians to clearly guide the clinicians in their management of patients and in their antimicrobial stewardship. Finally, other biomarkers (from hosts, host immune responses, wounds) identified by metaproteomic or metabolomic approaches could represent an attractive solution in the future [79].

## Conclusions

Current challenges for non-surgical chronic wound management include decreasing the delay in microbial identification of wound colonisation. However, the distinction between normal colonisation and infection remains unclear, leading to overtreatment, which in turn contributes to the increase in multidrug resistance. Moreover, biofilm formation following wound colonisation by pathogenic and commensal bacteria increases the risk of wound infection. The new metagenomics approaches represent a promising solution and could be implemented in future routine microbiology for the documentation of chronic wounds and the surveillance of post-treatment wound-colonising microorganisms [80, 81]. This review confirmed the need for standardised protocols to study chronic wound microbiota, including sampling methods, sample preparation, and DNA extraction. Future comparative investigation based on microbiomes from wounds, different parts of the body, and other environmental sources are needed to understand the origins of wound microbiota and its implications in wound evolution.

### Electronic supplementary material

Below is the link to the electronic supplementary material.

Supplementary Material 1

Supplementary Material 2

Supplementary Material 3

Supplementary Material 4

Supplementary Material 5

## Data Availability

All data generated or analysed during this study are included in this published article and its supplementary information file.
