# Supplementary material for: Direct metagenomics investigation of non-surgical hard-to-heal wounds: a review
Source: Ann Clin Microbiol Antimicrob. 2024 May 3;23:39. doi: 10.1186/s12941-024-00698-z (PMC11069288; doi:10.1186/s12941-024-00698-z)
Supplement: Supplementary file 1 — Supplementary Material 1 [file 12941_2024_698_MOESM1_ESM.docx]

**Supplementary data**


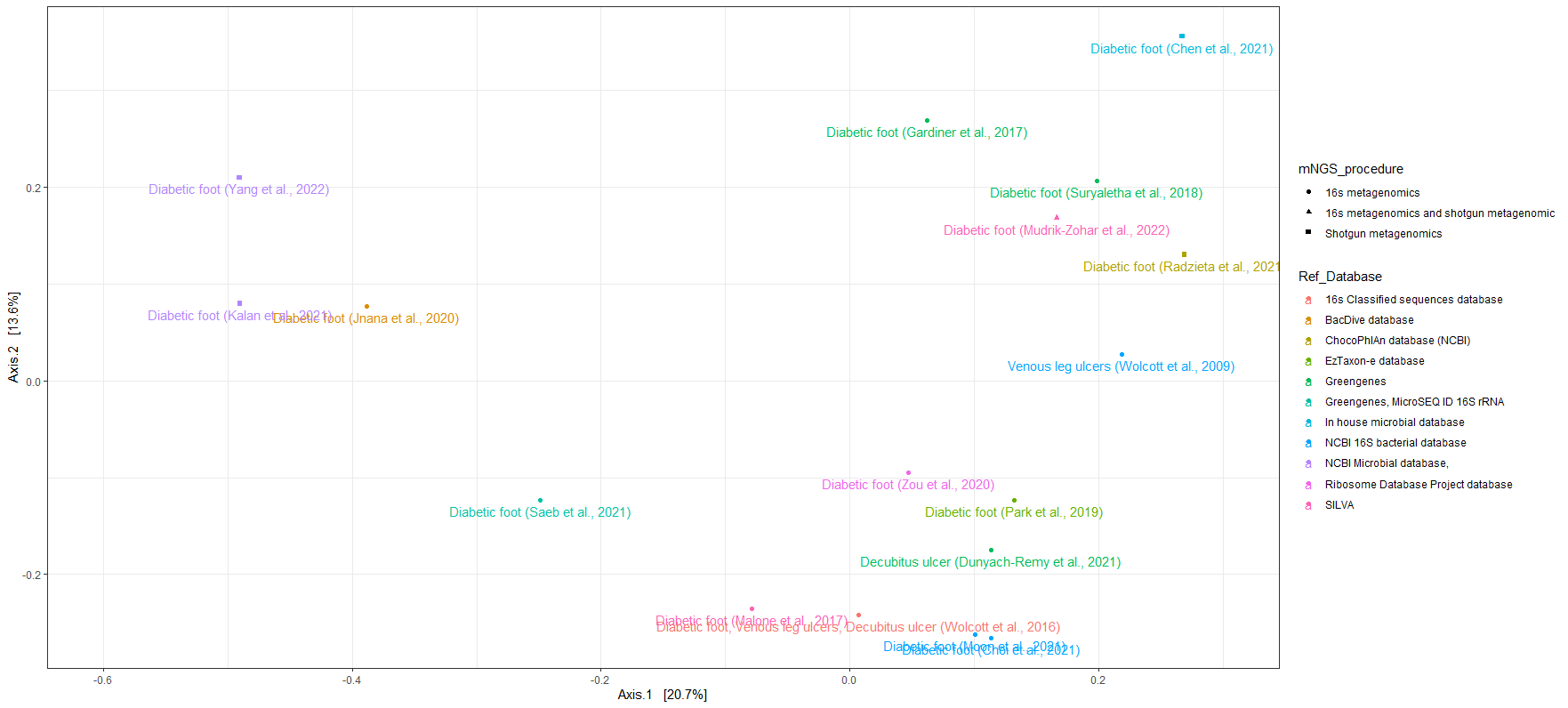


**Supplementary Figure 1**: Distribution of the published studies according to the metagenomics sequencing process and the used database.


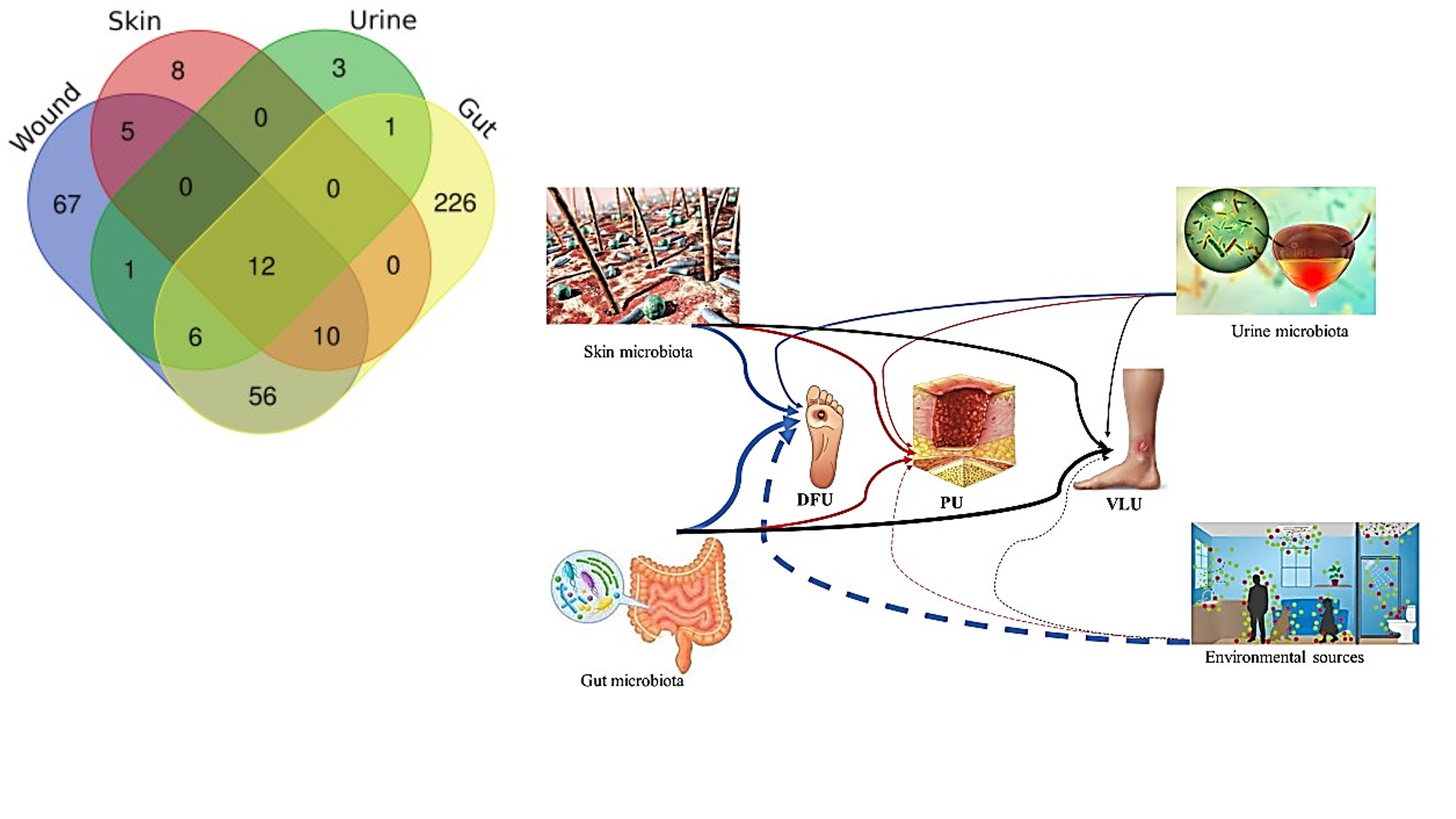


**Supplementary Figure 2**: Different origins of the bacterial species identified on chronic wounds. A Venn diagram represents the microorganisms shared in the different chronic wounds (DFU, diabetic foot ulcer; PU, pressure ulcer; VLU, venous leg ulcer) and their origins (wounds, skin, urine, gut). The linen's thickness corresponds to the number of shared microbes.


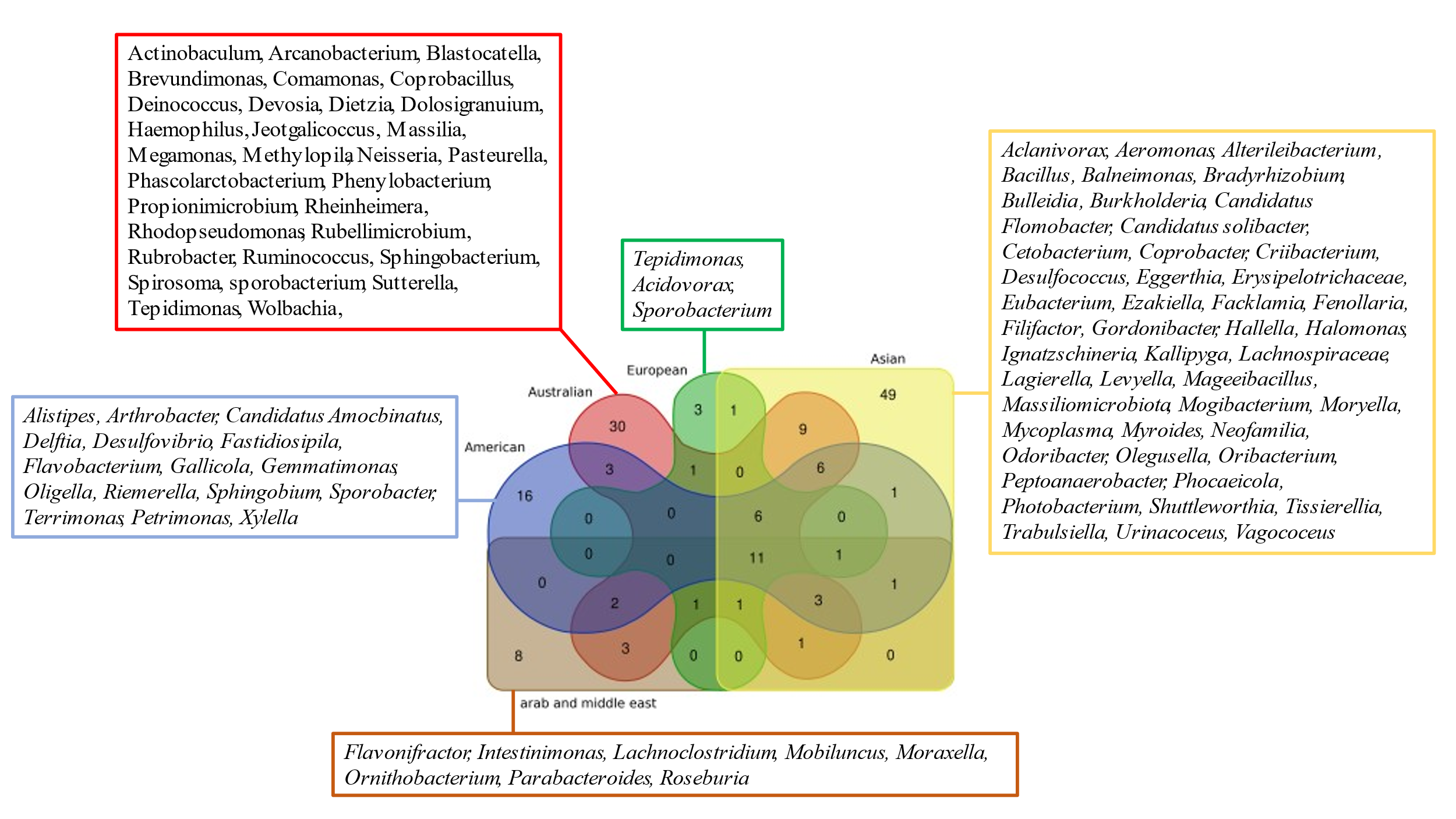


**Supplementary Figure 3**: Venn diagram showing the distribution of the microbial species between according to geographical location.
